# Induced Torpor as a Countermeasure for Low Dose Radiation Exposure in a Zebrafish Model

**DOI:** 10.3390/cells10040906

**Published:** 2021-04-14

**Authors:** Thomas Cahill, Willian Abraham da Silveira, Ludivine Renaud, Tucker Williamson, Hao Wang, Dongjun Chung, Ian Overton, Sherine S. L. Chan, Gary Hardiman

**Affiliations:** 1 School of Biological Sciences & Institute for Global Food Security, Queens University Belfast, Belfast BT9 5DL, UK; tcahill01@qub.ac.uk (T.C.); W.daSilveira@qub.ac.uk (W.A.d.S.); hwang24@qub.ac.uk (H.W.); 2 Department of Medicine, Medical University of South Carolina, Charleston, SC 29425, USA; renaudl@musc.edu; 3 Department of Drug Discovery and Biomedical Sciences, Medical University of South Carolina, Charleston, SC 29425, USA; tjw2219@gmail.com (T.W.); chans@musc.edu (S.S.L.C.); 4 Department of Biomedical Informatics, The Ohio State University, Columbus, OH 43210, USA; chung.911@osu.edu; 5 Patrick G Johnston Centre for Cancer Research, Queen’s University Belfast, Belfast BT9 7AE, UK; I.Overton@qub.ac.uk

**Keywords:** torpor, countermeasure, space, radiation, zebrafish, metabolism, temperature, signalling pathways, transcriptome, bioinformatics

## Abstract

The development of the Artemis programme with the goal of returning to the moon is spurring technology advances that will eventually take humans to Mars and herald a new era of interplanetary space travel. However, long-term space travel poses unique challenges including exposure to ionising radiation from galactic cosmic rays and potential solar particle events, exposure to microgravity and specific nutritional challenges arising from earth independent exploration. Ionising radiation is one of the major obstacles facing future space travel as it can generate oxidative stress and directly damage cellular structures such as DNA, in turn causing genomic instability, telomere shortening, extracellular-matrix remodelling and persistent inflammation. In the gastrointestinal tract (GIT) this can lead to leaky gut syndrome, perforations and motility issues, which impact GIT functionality and affect nutritional status. While current countermeasures such as shielding from the spacecraft can attenuate harmful biological effects, they produce harmful secondary particles that contribute to radiation exposure. We hypothesised that induction of a torpor-like state would confer a radioprotective effect given the evidence that hibernation extends survival times in irradiated squirrels compared to active controls. To test this hypothesis, a torpor-like state was induced in zebrafish using melatonin treatment and reduced temperature, and radiation exposure was administered twice over the course of 10 days. The protective effects of induced-torpor were assessed via RNA sequencing and qPCR of mRNA extracted from the GIT. Pathway and network analysis were performed on the transcriptomic data to characterise the genomic signatures in radiation, torpor and torpor + radiation groups. Phenotypic analyses revealed that melatonin and reduced temperature successfully induced a torpor-like state in zebrafish as shown by decreased metabolism and activity levels. Genomic analyses indicated that low dose radiation caused DNA damage and oxidative stress triggering a stress response, including steroidal signalling and changes to metabolism, and cell cycle arrest. Torpor attenuated the stress response through an increase in pro-survival signals, reduced oxidative stress via the oxygen effect and detection and removal of misfolded proteins. This proof-of-concept model provides compelling initial evidence for utilizing an induced torpor-like state as a potential countermeasure for radiation exposure.

## 1. Introduction

### 1.1. Expanding the Human Footprint

The first crewed Apollo mission to the moon in 1969 represented a historical milestone in scientific achievement and human exploration, taking humans on a 3-day journey to our closest celestial neighbour. This endeavour faced massive challenges including engineering of the Space Launch System, performing a safe landing on the lunar surface and programming electrical systems using relatively primitive computational power compared to today’s standards. Over a half century later NASA announced its plans to land the first woman on the moon by 2024, and to develop a sustainable lunar presence by 2028 involving the use of a manned lunar orbiter that will serve as gateway to the surface with lunar landers. Our presence on the moon will serve as a testing bed to develop new technologies that will extend our footprint across the solar system, eventually taking us to Mars in the 2030s [1]. While technology and computing power is much more advanced today, returning to the moon and onward to Mars present a new set of obstacles. 

### 1.2. The Challenges of Spaceflight

Mars, at its nearest distance from Earth is much further than the moon at between 57.6 million or 249 million km depending on the alignment with Earth. A Mars mission that takes advantage of close orbital alignments would involve a ~200 day transit there, a proposed ~500 day stay and a ~200 day return journey [2]. The long duration of these missions with increased distance from earth and prolonged exposure to environmental stressors impose new technical and physiological challenges that must be countered to warrant a successful mission while ensuring crew health and safety. Two of the key obstacles for long-term space exploration include accessibility of food, water and oxygen and exposure to high radiation levels [3,4]. Accessibility of food, water and oxygen becomes challenging for longer term interplanetary travel as resupply missions are made difficult by the changing orbital distances between planets. These missions must therefore be less dependent on Earth. They will require provision for enough resources for the duration of the mission. Alternatively technologies that can mitigate the need for resupplies must be developed [5]. Studies evaluating the efficacy of different shielding materials against space radiation have found secondary radiation is produced upon impact and can contribute to radiation exposure. While these studies revealed that lower atomic mass materials such as hydrogen reduced the number of secondary radiation particles, shielding poorly attenuates high energy galactic cosmic rays [6].

Therefore, emphasis needs to be placed on developing viable countermeasures against the harmful effects of ionising radiation during space travel. At the cellular level, irradiated cells enter the DNA damage-dependent cell cycle arrest in an attempt to re-establish chromosome integrity [7]. Ionising radiation also produces short-lived free radicals including reactive oxygen and nitrogen species as well as changes in redox signalling linked to disruption of metabolic processes that persist long after the radiation exposure [8]. 

### 1.3. Effects of Radiation on the GIT

Cells of the GIT are particularly radiosensitive, a quality arising from their rapid proliferation rate [9]. The key event in the pathophysiology of intestinal radiation toxicity is enterocyte depletion, with possible vascular damage at higher radiation doses [10]. In the longer term, tissue remodelling after the radiation damage alters the structure, motility and absorption of the gut, while fibrosis renders it more rigid and susceptible to adhesions, stenosis and perforation [11,12]. These changes can alter GIT functionality and increase susceptibility to disease-causing nutrient deficiencies. Moreover, the principal effect of radiation exposure is an increased risk of cancer [13]. This highlights the emphasis that should be placed on improving our understanding of the risks of radiation on astronauts’ health to develop appropriate, viable and innovative countermeasures in tackling the outstanding challenges of space travel.

### 1.4. Torpor

In mammals, torpor is characterised by lowering the metabolic rate and body temperature, which results in a reduced need for nutrients, oxygen and water [14,15]. The artic ground squirrel for example, achieves torpor by lowering its body temperature to −3 °C and simultaneously decreasing its metabolic rate by approximately 90% [16]. Hibernating animals utilise this state as a survival mechanism to conserve energy during periods of food scarcity or cold temperatures and has therefore been associated with conferring a survival advantage. The idea that hibernation confers protective effects prompted studies of radiation exposure in hibernating squirrels which showed that irradiated hibernating squirrels had increased mean survival times compared to active controls however the molecular mechanisms remain to be elucidated [17]. The induction of an induced torpor-like state in humans, which has been suggested as a countermeasure for space travel as far back as the 1960s [18] would therefore effectively address both of the challenges outlined above, by minimising nutrient, O_2_ and H_2_O consumption for long-term space travel and protecting against radiation damage [3,19]. Interestingly, a recent study of the bones of early humans shows evidence of seasonal variation in bone growth and patterns of lesions that are consistent with those found in hibernating mammals indicating that our ancestors may have adapted to hibernating during extreme cold periods [20]. While modern humans have ceased to employ hibernation as a survival mechanism, controlled therapeutic hypothermia has been used in medicine to successfully lower mortality rates and improve the neurological function of patients that have suffered from acute trauma, cardiac arrest or stroke [21]. This highlights the conferred protective effects and their application to broader human health and wellbeing and supports the viability of induced torpor during space travel. However, further work is needed to explore cooling methods and the effects of maintaining hypothermia long term.

### 1.5. Induced Torpor as a Countermeasure for Low Dose Radiation Exposure in a Zebrafish Model

Our overarching hypothesis is that induced torpor could minimise the damage caused by exposure to space radiation. To test this hypothesis, the body temperature and metabolism of zebrafish (*Danio rerio)* were lowered via ambient temperature reduction coupled with the addition of melatonin to induce a torpor-like state. The fish were exposed to low dose radiation (~0.3 Gy) similar to what would be experienced on a 6-month journey to Mars to allow assessment of the potential protective properties of torpor. More specifically, transcriptomic profiling enabled exploration of the implications that space travel might have on GIT functionality. Zebrafish were used in a model of induced torpor as they are poikilothermic and vary their body temperature based on their ambient temperature by modulating their gene expression and metabolism [22,23]. While they have been found to inhabit a range of temperatures, 28.5 °C has been cited as the optimal temperature [24]. Additionally, Malek, et al., [25] detailed the reduction and maintenance of the effect of reducing ambient temperatures from 28 °C to 18 °C for 1 year. Studies have shown that a reduction of 10 °C in body temperature in heterotherms reduces their metabolic rate by 50% of basal levels [19]. Moreover, zebrafish were also chosen as the modulatory neurotransmitters in their brain are similar to humans; exposure to a wide range of concentrations (10–100 nM) of the hormone melatonin promotes a sleep-like state, reducing locomotor activity and elevating the arousal threshold [26]. While melatonin is a potent antioxidant and regulator of the circadian rhythm [27], it was used primarily to increase sedation and reduce activity, to replicate the inactivity seen in hibernating animals. Zebrafish further offer unique advantages as a model organism as approximately 70% of their genes are orthologous with human genes, 84% of which are implicated in human disease [28]. Finally, the availability of a high-quality reference genome makes it an ideal model for rapid genomic and phenotypic assessment [29]. 

## 2. Materials and Methods

### 2.1. Zebrafish Husbandry

The zebrafish (AB strain) were obtained from the Zebrafish International Resource Centre and maintained and crossed according to standard housing methods. Adult zebrafish were housed at a maximum 6 fish per 1 L glass beaker with lid (allowing air flow but preventing fish mortality from jumping out of the beaker) in an incubator at 28.5 °C with a light cycle of 14 h ON (light) and 10 h OFF (dark). Zebrafish were fed Gemma Micro 300 standard diet every other day (Skretting, Westbrook, ME, USA) early in the morning, and 20 min after feeding, debris was aspirated from the beaker bottom and 75% of the water was changed using reservoir water (Reverse Osmosis water supplemented with Instant Ocean salts, sodium bicarbonate and Stress Coat, maintained at pH 7.4) to increase water life support capability. Beakers used to house fish were previously cleaned and autoclaved. All procedures were performed in accordance with The Medical University of South Carolina (MUSC), Institutional Animal Care and Use Committee (IACUC) guidelines (IACUC-2018-00278). All animals were treated humanely and with regard for alleviation of suffering. 

### 2.2. Locomotion Assay for Activity Score

A locomotion assay analysis was employed to determine if the addition of melatonin and the reduction of ambient temperatures induced a resting-state with reduced movement. Locomotor activity in the zebrafish was therefore recorded using a Basler acA1300-60 gm GigE camera and tracked using the movement tracking Ethovision XT 14 software (Noldus Information Technology, Inc., Leesburg, VA, USA) [30]. Briefly, one zebrafish was transferred to a new beaker with water in a temperature that matched its previous beaker, after allowing 5 min to acclimatise, a 15-min video was recorded. The EthoVision software tracked the fish movement to calculate the total distance travelled and to determine the regions the animal occupied the most. Videos of swimming behaviour recorded for the locomotion assay along with raw data showing distance moved and time spent in different zones, that were used to calculate the activity score can be found in the Supplementary Materials (supplemental video Trial 1—12.mp4 and Supplementary Table S1, respectively). A combination of active swimming time, and time spent at the bottom or at the upper part of the beaker was used to calculate an activity score for the fish allowing for behavioural profiling of the animals as outlined by Nishimura et al. [31]. Hence, a higher activity score indicates a more balanced use of space, with more frequent movement between the bottom and upper part of the beaker, whereas a lower activity score indicates less movement. An ANOVA test was performed to test for statistical differences between the groups with subsequent post-hoc testing using Tukey’s HSD. 

### 2.3. Development of the Induced Torpor Model and Radiation Protocol

**Figure 1:**
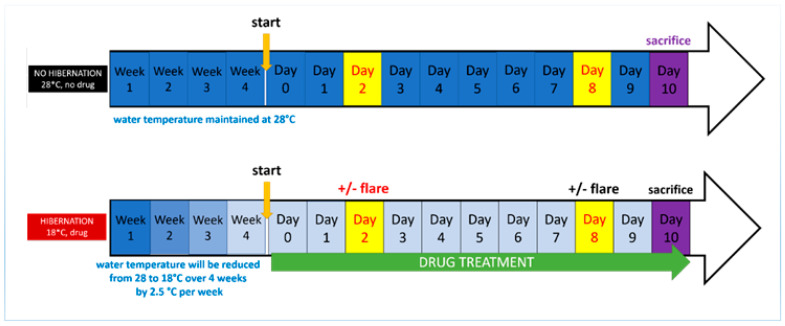
Experiment timeline shows the radiation group (**top**) maintained at 28.5 °C, receiving radiation on the 2nd and 8th day prior to sacrifice on the 10th day. The torpor + radiation group (**bottom**) was lower temperature acclimatised over 4 weeks, when 18.5 °C was reached they also received 24 µM of melatonin daily for 10 days. Radiation exposure was administered on the 2nd and 10th day prior to sacrifice on the 10th day.

Developing models of induced torpor and radiation exposure required a series of experimental groups described in Table 1 with different variables to assess their effects and facilitate comparisons. A control group was maintained at an ambient temperature of 28.5 °C (28.5-Ctrl). A melatonin group (28.5-mel) received melatonin daily for 10 days at a concentration of 24 µM to reduce locomotion and arousal. To maintain levels of melatonin and protect against metabolism and degradation [32], 75% of the water was replaced daily with a mixture of saline water and melatonin. Melatonin was purchased from Sigma-Aldrich (St. Louis, MI, USA) with ≥98% purity, it was maintained at −20 °C in powder and dissolved in DMSO prior to use. A reduced temperature group (18.5-Ctrl) was kept at 18.5 °C to decrease their metabolism. The reduction in ambient temperature was carried out in weekly decrements of 2.5 °C over the course of 4 weeks to avoid thermal shock, as previously described [25]. An induced torpor group (18.5-mel) was maintained at 18.5 °C with 24 µM melatonin. Again, fish were acclimatised over a 4-week period and melatonin was added for 10 days once the water temperature reached 18.5 °C. A radiation group was exposed to a total whole body dose of 32.68 cGy. Prior to radiation exposure adult zebrafish were anaesthetised with 0.02% tricaine. They were then placed one at a time on 60-mm × 15-mm Petri dishes containing water and placed atop a 3-inch spacer ready for irradiation. Radiation exposure occurred for 6 s at 163.40 cGy/min resulting in total exposure of 16.34 cGy. Fish were exposed to radiation on the 2nd and 8th day resulting in a combined whole-body dose of 32.68 cGy. Irradiation was carried out at MUSC in accordance with IACUC-2018-00278 using the following irradiator; Shepherd Model 143-68, Serial Number 8020, (JL Shepherd and Associates, San Fernando, CA, USA), with a Caesium 137 radiation source. After radiation exposure, fish were placed in a temporary tank free of tricaine to recover, and then transferred back into the main tank with other fish from the same experimental group. A torpor + radiation group was also established using a cold acclimatised group with melatonin that was also subject to the radiation protocol. Fish were sacrificed on the 10th day of the experimental timeline (starting from the end of the acclimatisation protocol), represented schematically in Figure 1. 

### 2.4. RNA Extraction and Sequencing

Total mRNA was extracted from GIT tissues using the miRNeasy Qiagen kit (Qiagen, Hilden, Germany). To prepare mRNA-Seq libraries the TruSeq RNA Sample Prep Kit (Illumina, San Diego, CA, USA) was utilised; 100–200 ng of total input GIT RNA was used in accordance with the manufacturer’s protocol. High through-put sequencing was performed at the University of North Carolina-Chapel Hill High Throughput Sequencing Facility, Chapel Hill, NC and the Queens University Belfast, Genomics Core Technology Unit. RNAseq was performed on Illumina Next SEQ 500 and HiSeq4000 instruments with each mRNA library sequenced to a minimum depth of 25 million reads. RNA-Seq data have been submitted to the NCBI Gene Expression Omnibus, accession number GSE169522.

### 2.5. RNA-seq Data Processing and Differential Expression

Sequence quality was assessed with FastQC [33] to identify the over-represented sequences and low-quality reads which were removed using Cutadapt [34]. The STAR aligner [35] was utilised to align the RNA-Seq reads to the zebrafish genome (GRCz11) and HTSeq [36] to determine the number of reads per transcript. DESeq2 [37] allowed the determination of differentially expressed (DE) genes which were subsequently used in downstream pathway analysis according to their absolute fold change (linear FC of 1.5, or log2FC of 0.58) and a FDR adjusted *p*-value (q ≤ 0.1), calculated using the Benjamini-Hochberg multiple testing adjustment procedure [38]. Hierarchical clustering and principal component analysis (PCA) plots were used to explore the patterns in sample variation. Additionally, Ensembl human orthology [39] was determined in order to leverage human annotation for more comprehensive data analysis in system level analysis which benefit from the greater annotation of human genes, as demonstrated by Huff et al., [40].

### 2.6. Experimental Validation

Validation of the genes outlined in Supplementary Table S2 was performed by means of qPCR. Briefly, a total of 0.5 µg of RNA from 28.5-ctrl, 18-mel, 28.5-rad and 18.5-mel-rad was used to synthesise cDNA employing the iScript Reverse Transcription Supermix (Bio-Rad). Next, two replicates were used in the quantification of gene expression performed using SYBR Green qPCR (ThermoFisher, Waltham, MA, USA) on a Roche LightCycler 480 Instrument II (Roche Diagnostics, Rotkreuz, Switzerland). Intron spanning primers (Supplementary Table S2) were designed using Primer-Blast [41]. The relative induction of gene mRNA expression was then calculated using *actn2b* expression for normalisation and values for the experimental groups were compared with values from the control group (28.5-ctrl). T-Tests were performed to test for significance and results were plotted using GraphPad Prism (San Diego, CA, USA).

### 2.7. Pathway Analysis

The differentially expressed (DE) zebrafish genes (q < 0.1, FC > 1.5) for the radiation (28.5-rad), torpor (18.5-mel) and torpor + radiation (18.5-mel-rad) group measured in comparison to the control group (28.5-Ctrl) were subject to over-representation with Webgestalt [42] as zebrafish and human using the respective gene symbols. Next pathway impact analyses were performed in iPathwayGuide (Advaita Bioinformatics, Ann Arbor, MI, USA) [43] using human gene symbols. Webgestalt was employed to define enriched gene ontologies and KEGG pathways (q ≤ 0.05) taking either up- or downregulated genes as input to gain insights on affected cellular processes. Enriched gene-ontologies and impacted pathways identified using iPathwayGuide were used to supplement the results taking advantage of a topology-based approach which considers the type, function, position and interaction between genes in each pathway to help reduce false positives. 

### 2.8. Network Analysis

A zebrafish functional gene network (ZFGN) was kindly provided by Olga Troyanskaya [44]. The top 500 upregulated and 500 downregulated genes (FC > ±1.5) for the radiation (28.5-rad) and torpor + radiation (18.5-mel-rad) were analysed by NetNC [45] in ‘Functional Target Identification’ (FTI) mode using the network formed from all ZFGN edges that had edge weight >0.5. The resulting networks for each condition were merged and subsequently visualised using Cytoscape v3.7.2 [46] and a gene ontology analysis was performed on distinct clusters using the Cytoscape plugin ‘BiNGO’ [47]. 

## 3. Results 

### 3.1. Temperature and Melatonin Reduce Zebrafish Activity

**Figure 2:**
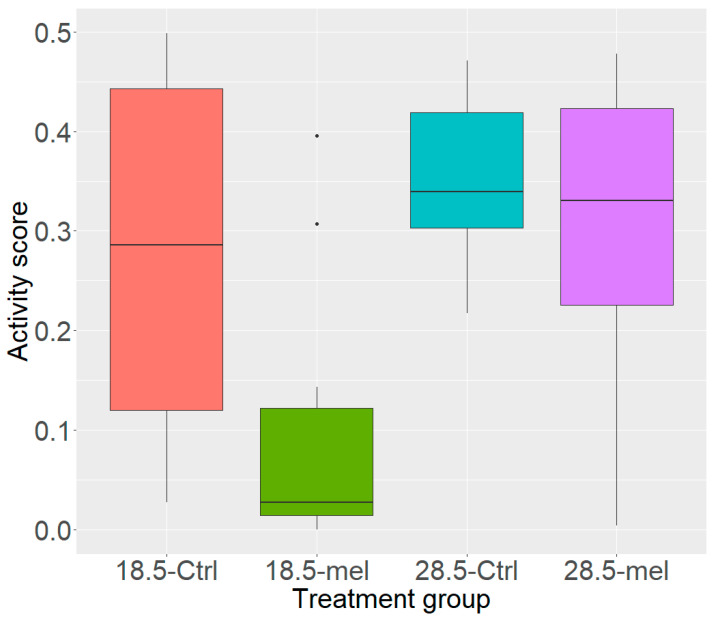
Activity Scores. The control group (28.5-Ctrl) inhabited all areas of the beaker. The torpor group (18.5-mel, q = 0.002) had a significantly lower activity score than either control or temperature group (28.5-Ctrl, 18.5-Ctrl, q = 0.030). Lowering temperatures alone (18.5-Ctrl, q = 0.030) reduced activity while the melatonin group (28.5-mel, q = 0.538) did not significantly reduce activity.

Reduced temperature alone (HSD q = 0.030) or in combination with melatonin (q = 0.002) significantly reduced the activity levels in zebrafish compared to the 28.5-Ctrl (Figure 2). However, melatonin alone did not lower the activity score significantly (q = 0.538). Our data validated the torpor-like model in zebrafish, showing that it is possible to increase the resting phase exploiting the combination of melatonin treatment, and reduced temperature. Raw video data are available in Supplemental Material (Supplementary Videos S1–S12) showing swimming behaviour for the fish from each experimental group.

### 3.2. Transcriptomic Characterisation of Induced Torpor Model Reveals a Reduction in Metabolism

Differential expression analysis on the transcriptomic data of the torpor group (18.5-mel vs 28.5-Ctrl) revealed the upregulation of 3602 genes and the downregulation of 2413 genes in the GIT (q ≤ 0.1, FC ± 1.5) (Supplementary Table S3). Over-representation analysis (ORA) and impact analysis are found in Supplementary Tables S4–S6.

**Figure 3:**
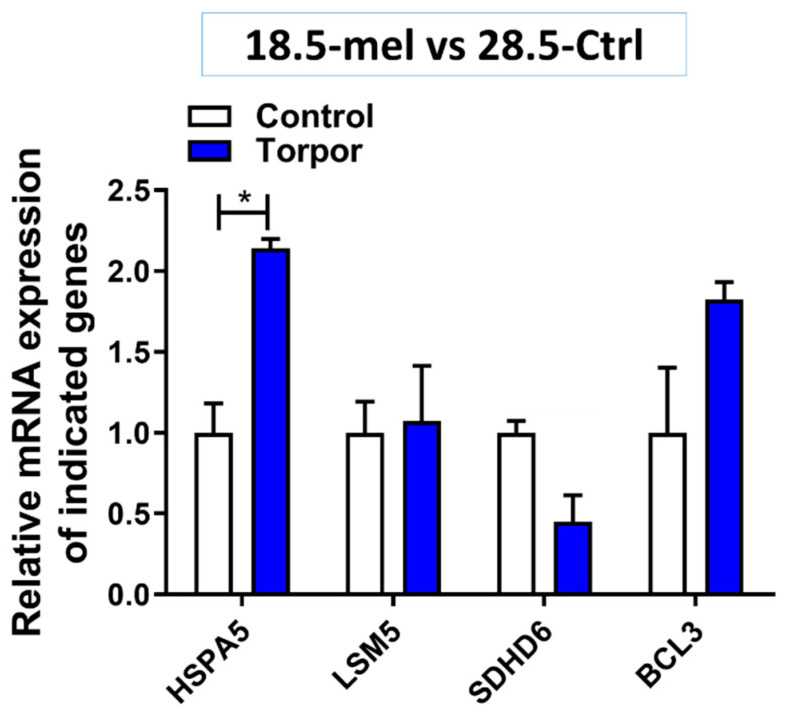
Gene expression in the torpor (18.5-mel) group compared to that in the control (28.5-Ctrl) group. The figure shows the relative increase in expression of *HSPA5* (*p* = 0.013) and while not significant *BCL3* expression (*p* = 0.107) is also increased. It also demonstrates an almost significant decrease in expression of SDHD6 (*p* = 0.05) compared with control, supporting the results from DE analysis. * *p* < 0.05.

The results suggest that the induced torpor leads to an upregulated mitosis with an increased expression of chromosome organisation genes (GO:0051276), cyclins (*CCNB1, CCNL1, CCNT1*) and cyclin-dependent kinases (*CDK11B, CDK12, CDK13*) which drive progression through the cell cycle [48]. In agreement, they also show both an increase in pro-survival signals including *BCL3* [49] and *TRAF1/2* [50] as well as a decrease in pro-apoptotic signals (*Casp8*, *Casp10*) [51]. Perturbation analysis also showed a decrease in pro-apoptotic genes such as *Bid* and *TRAILR2*, as well as an increase in *Bcl-2*, a pro-survival gene [52]. Quantification of gene expression was performed on *BCL-3* using qPCR: and while not significant (*p* = 0.107), the expression levels as seen in Figure 3 agree with DE analysis which showed a significant upregulation (q 1.65 × 10^−15^) compared to the control, supporting the idea of anti-apoptotic signals.

We reported an increase in RNA splicing (GO:0008380, GO:0000375, GO:0006397), with an upregulation of transcripts encoding spliceosome subunits (*CACTIN*) [53], and associated spliceosome proteins (*CWC22, CWC25*) [54,55]. Additionally, several GO terms relating to protein targeting to the ER (GO:0045047) were enriched with an overall decrease in expression for these pathways. However, genes involved in detecting misfolded proteins (*HSPA5*) [56] and targeting for degradation (*DERL2, UBXN4*) [57,58] were upregulated as seen in Figure S2. Quantification of *HSPA5* in qPCR also showed a relative increase in expression (*p* = 0.013) compared with the control (28.5-Ctrl) as seen in Figure 3.These pathways, namely, RNA splicing [59], protein folding and mitosis are also found to be adaptive response in cold acclimatised fish such as the Antarctic toothfish [60]. 

**Figure 4:**
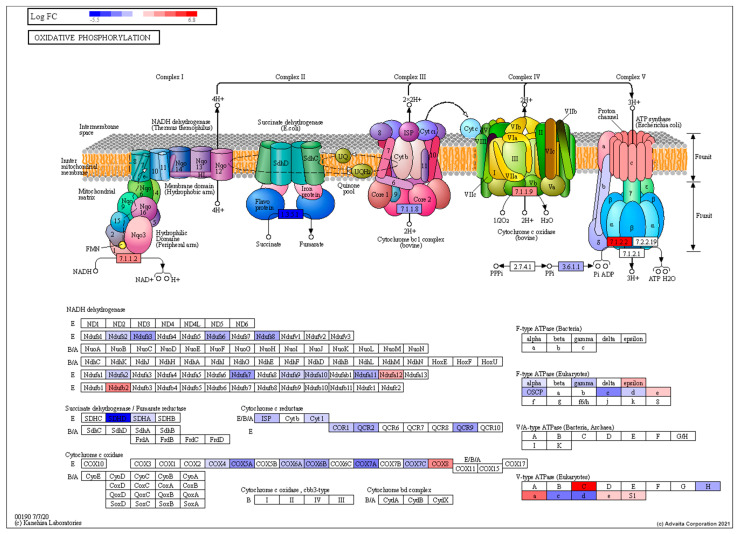
Pathway topological diagram of the oxidative phosphorylation pathway produced by KEGG showing differentially expressed genes from a comparison between the torpor and control group showing the torpor group experienced an overall downregulation of several key subunits of electron transport chain proteins such as NADH dehydrogenase, succinate dehydrogenase and cytochrome c oxidase, indicating reduced energy production. RED: upregulated, BLUE: downregulated.

As expected, torpor also reduced the metabolism of key macronutrients such as lipids (GO:0006629) and proteins (GO:0006518) with a decrease in expression of key lipolytic (*ACSL5*, *DGAT2*) [61,62] and proteolytic (*DPP4*) [63] genes. Similarly, we found downregulation of a number of genes involved in metabolic pathways (Supplementary Figure S1) including glycolysis (*GAPDH*) [64], the tricarboxylic acid (TCA) cycle (*SDHD, ACO1* and *IDH2)* [65,66,67] and oxidative phosphorylation (*Ndufs3*, *SDHD* and *Cyt1*) [65,68,69] (shown in Figure 4). In agreement with the DE analysis, *sdhd6* quantification in qPCR showed a relative decrease (*p* = 0.05) in expression compared with the control (28.5-Ctrl) (Figure 3). Our analysis also points to a decrease in lipid, glucose and amino acid uptake with downregulation of specific transporters such as *CD36, SLC5A1* and *SLC6A19* [70,71,72], respectively. Furthermore, a decrease in protein synthesis was suggested by the downregulation of ribosomal subunits including *RSP20, RSL3, RPL4, RPS3, RPL23* [73]. 

### 3.3. Low Dose Radiation Affects Metabolism and Absorption in the GIT

**Figure 5:**
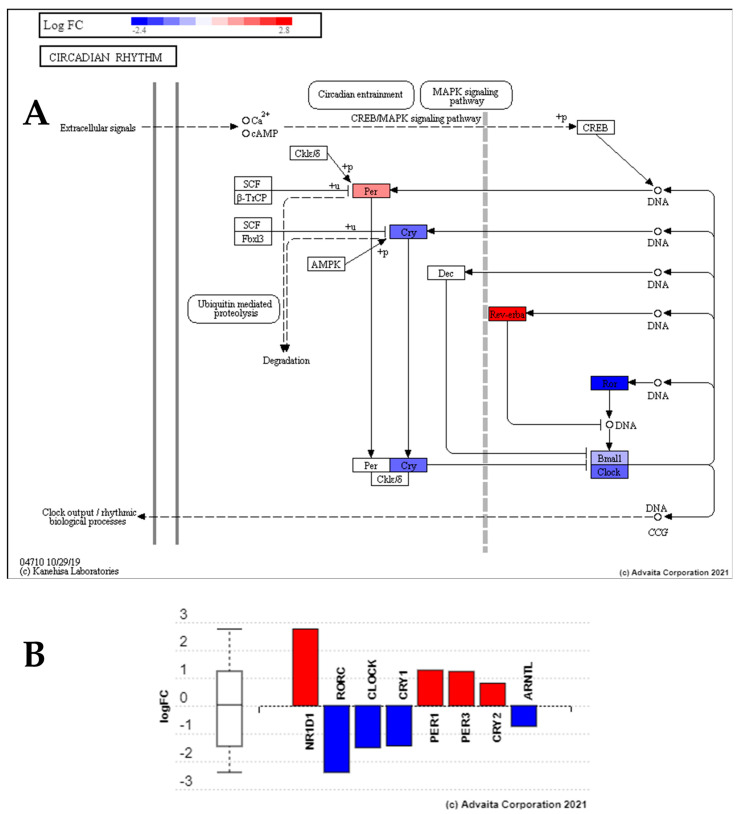
Perturbation of circadian rhythm pathway genes from exposure to low dose radiation compared to control group. This reveals upregulation of Per and Rev-Erba, and the downregulation of Cry, Clock, Bmal and Arntl in comparison to the control group (28.5-Ctrl), suggesting low dose radiation perturbs the circadian rhythm. (**A**) Pathway topological diagram showing position and regulation of differentially expressed circadian rhythm genes. RED: upregulated, BLUE: downregulated. (**B**) Bar chart showing fold change of differential expression of the key clock genes.

We compared the radiation (28.5-rad) and control group (28.5-Ctrl) to define how low dose radiation, as may be experienced during long-term space travel (~0.3Gy), affects the GIT. Differential expression analysis revealed that 146 genes (q ≤ 0.1) were significantly impacted, 99 upregulated, and 47 were downregulated compared to the control group (28.5-Ctrl) (Supplementary Table S7). ORA in Webgestalt and impact analysis in iPathwayGuide results can be found in Supplementary Table S8–S10. They revealed that low dose radiation affected stress-related pathways such as circadian rhythm (GO:0048511, hsa04710) (Figure 5), and glucocorticoid receptor signalling pathway (GO:2000322) with several shared genes dysregulated in each pathway (*CLOCK, CRY1, PER1, CRY2* and *ARNTL*) [74]. Cell cycle changes were suggested by the negative regulation of DNA transcription (GO:1903507) and RNA biosynthesis (GO:1902679), indicating growth arrest, a response typical of radiation exposure used to repair damage and prevent transduction of DNA mutations to daughter cells [75]. 

**Figure 6:**
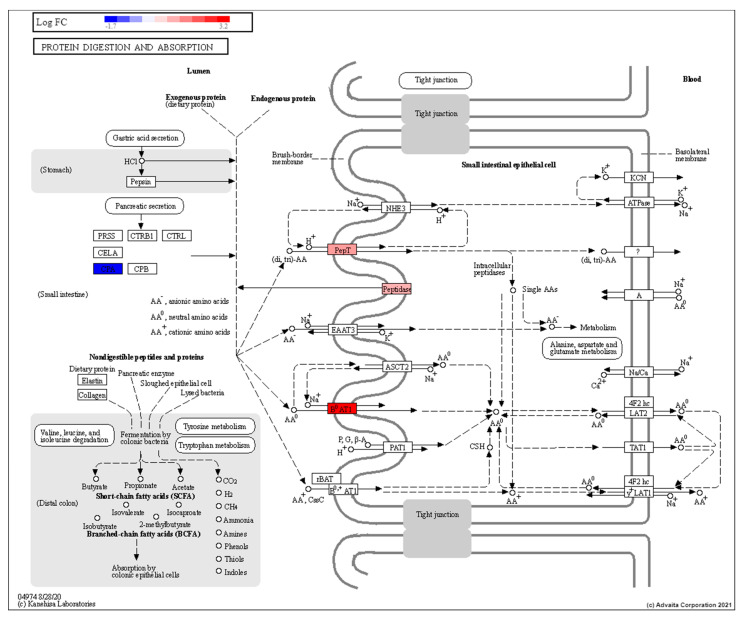
Topological diagram of the protein digestion and absorption pathway showing the upregulation (28.5-Rad vs 28.5-Ctrl) of peptidases and amino acid transporters on the brush border membrane in response to low dose radiation exposure RED: upregulated, BLUE: downregulated.

**Figure 7:**
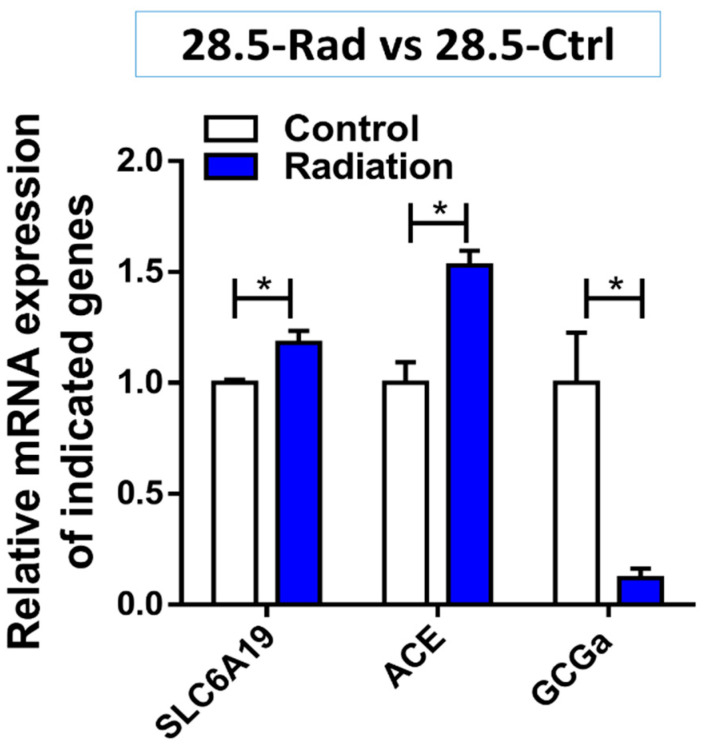
Gene expression in the radiation (28.5-Ctrl) group compared with control (28.5-Ctrl). The figure shows the increase in expression of *SLC6A19* transporter (*p* = 0.041) and the angiotensin system gene *ACE* (*p* = 0.022) while *GCGa* involved in increasing blood sugar levels is downregulated (*p* = 0.032) compared to the control. * *p* < 0.05.

Results point to radiation-induced changes to protein digestion and absorption (GO:0043171, hsa04974) (as seen in Figure 6) involving peptidase enzymes (*DDP4)* which have previously been reported to be induced by low dose radiation exposure [76]. Upregulation of *SLC15A1* and *SCL6A19* also supports increased in amino acid uptake [76,77,78]. Similarly, these genes may also affect the balance of anions/cations such as chloride, bicarbonate or sodium [77]. Moreover, the genes involved in the renin-angiotensin system (RAS) including *ACE, ACE2* and *ANPEP* were upregulated and have been implicated in bicarbonate secretion, absorption of sodium, water and glucose absorption as well as digestion of peptides and secretion [79]. Validation of an increase in expression levels of *SLC6A19* (*p* = 0.041) and *ACE* (*p* = 0.022) by qPCR (Figure 7) support the DE analysis and the theory of radiation-induced changes to absorption and secretion. 

Increased lipid transport is also indicated by the upregulation of *ALP*, involved in the regulation of lipid absorption in the GIT [80] as well as increased transporter activity involving *ABCB1, ABCD4*, *ABCC4* and *STRA6* which contribute to barrier function, lipid transport [81], bile secretion [82] and have been linked to excess sodium loss, diarrhoea, dehydration and death after irradiation [83].

The results also suggest that low dose radiation is reducing glucose availability in the GIT due to the downregulation of *GCK* and *DLAT* involved in glycolysis [84]. A glucagon precursor (*GCG*) is also downregulated which plays a role in increasing blood sugar levels. The downregulation of *GCGa* (*p* = 0.032) was also validated using qPCR (Figure 7). Similarly the upregulation of *SREBF1* which is known to modulate insulin sensitivity [85] is in line with the literature showing that radiation exposure can lead to insulin resistance [86]. In addition, the upregulation of *KLF7* might suggest a decrease in insulin secretion [87]. This is consistent with previous work that demonstrated intestinal radiation exposure leads to a decrease in both glucose absorption and the glucose transporter proteins, GLUT4 and SGLT1 [88]. 

### 3.4. Pathway Analysis of Induced Torpor with Radiation Reveals Stress Response with Pro-survival Signals

**Figure 8:**
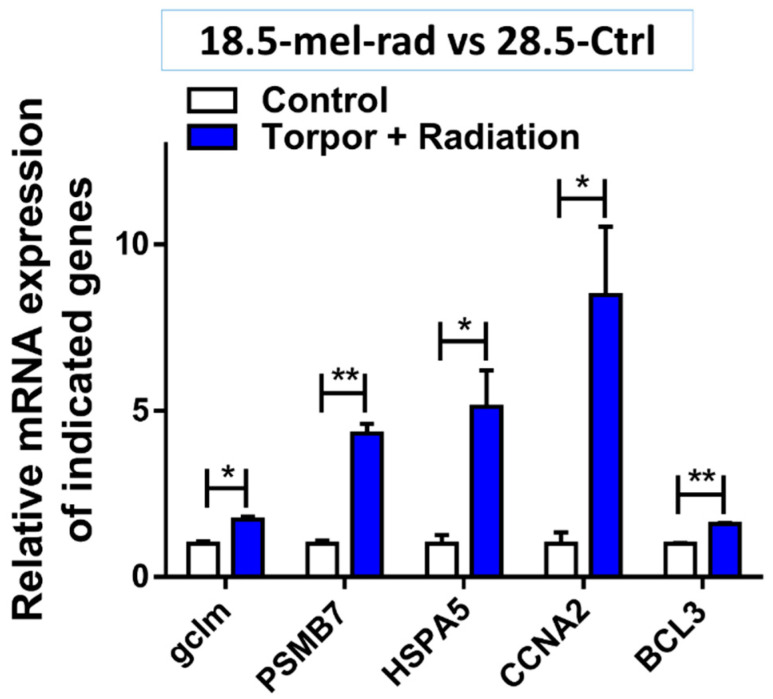
Validation of gene expression in the torpor + radiation (18.5-mel-rad) group compared with control (28.5-Ctrl). The figure shows the increase in expression of *GCLM* (*p* = 0.011), *PSMB7* (*p* = 0.004), *HSPA5* (*p* = 0.035), *CCNA2* (*p* = 0.036) and *BCL3* (*p* = 0.001911), relative to the control (28.5-Ctrl) involved in glutathione metabolism, proteasome complex, protein refolding, cell cycle and anti-apoptosis, respectively. * *p* < 0.05, ** *p* < 0.001.

We compared the torpor + radiation group (18.5-mel-rad) with the control group (28.5-Ctrl) and noted 1436 genes DE genes (q ≤ 0.1, FC ± 1.5), 749 upregulated and 690 downregulated relative to the control group (Supplementary Table S11). ORA and impact analysis results are in the Supplementary Tables S12–S14. Like that of the radiation group, the results indicate a response to stress (GO:0006950) which involved p53-mediated growth arrest (GO:0072331, GO:0000075), steroid hormone signalling (GO:0048545) and ferroptosis: programmed cell death caused by cytotoxic levels of lipid peroxidation [89]. We also observed upregulation of the immune response (GO:0002446) involving activation of the inflammatory NF-kB signal transduction pathway (GO:0038061) and release of pro-inflammatory cytokines such as TNF (GO:0033209). Moreover, glutathione metabolism was also indicated with an increase in expression of genes such as *gclm* (*p* = 0.011)*,* an increase of which was validated using qPCR, suggesting a response to oxidative stress (Figure 8).

**Figure 9:**
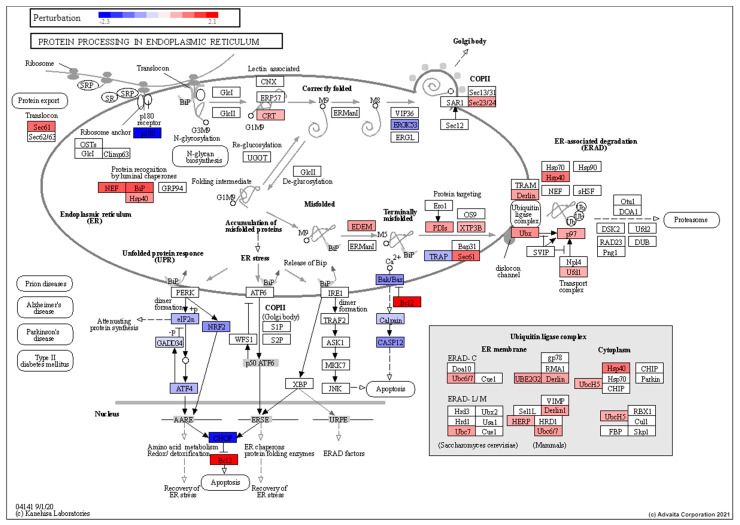
Perturbation of genes encoding proteins involved in ER-associated protein degradation showing an increase in expression of chaperones involved in detecting misfolded proteins (*NEF, BiP, Hsp40*), as well as those involved in the ER-associated degradation pathway (*Derlin, UBx*). This also shows an increase in anti-apoptotic genes (*BCL2*) and a decrease in pro-apoptotic genes (*Casp12*). RED: upregulated, BLUE: downregulated.

In line with the growth arrest, the halting of protein synthesis was also suggested from the downregulation of ribosomal subunits as well as the upregulation of *SRP9* which has previously shown to be essential for elongation arrest [90]. Like that of the torpor group we also seen the enrichment of genes involved in the ER-associated degradation pathway (GO:0036503) including the proteasome (GO:0010498) with a notable increase in expression of ubiquitin ligase complex genes, as seen in Figure 9. Moreover, validation of an increase in expression in *PSMB7* (*p* = 0.004) and *HSPA5* (*p* = 0.035) via Q-PCR as shown in Figure 8 support the idea of detection and removal of misfolded proteins in the ER.

We also see results such as an increase in mitosis (GO:1902850, GO:0044772, GO:0007059) and anti-apoptotic signals with the upregulation of anti-apoptotic genes, *BCL3.* Similarly, detection of increased levels of gene expression in qPCR of genes involved in cell cycle progression (*CCNA2*, *p* = 0.036) and anti-apoptosis (*BCL3*, *p* = 0.001) (Figure 8) reinforce the theory that torpor confers pro-survival signals, and continue too during stress. Again, we also see the enrichment of RNA splicing (GO:0008380) considered to be induced in a temperature-dependent manner [91]. 

Regarding metabolism, the combination of torpor and radiation exposure also produced mixed results. As seen in supplementary Figure S3, results like that seen in the radiation group demonstrate indications of an increase in lipid metabolism (GO:0055088) and biosynthesis (hsa00061) as reserves that are mobilised during stress. Similarly, the Peroxisome pathway is enriched, which is known to play a role in fatty acid oxidation [92]. However, given the torpor variable, results also show a decrease in glucose metabolism (GO:0006109), TCA cycle and oxidative phosphorylation (hsa01100). In addition, we noted downregulation of the metabolism of substrates such as amino acids (GO:0006520), coenzymes (GO:0006732), aldehydes (GO:0006081), ketones (GO:0042180) and esters (GO:0046434). 

Our findings also showed a response to hypoxia (GO:0001666), consistent with decreased oxygen and a reduced metabolism. The results additionally point to changes in GIT absorption with an upregulation of ABC transporters and those involved in mineral absorption of the SLC family (hsa04978) suggesting that torpor does not mitigate changes to absorption or secretion. 

A meta-analysis was performed in Advaitas iPathwayGuide [43] to find genes shared between the radiation group (28.5-Rad vs 28.5-Ctrl) and torpor + radiation group (18-mel-rad vs 28.5-Ctrl) with a focus on those that were differentially regulated between the two experimental groups. Genes including *PSMD3*, *TXN*, *GSTP1* and *HMGB1&3* were downregulated in the radiation group and upregulated in the torpor + radiation groups. *PSMD3* is a component of the proteasome involved in removing damaged or unfolded proteins, consistent with the reported enrichment of ER-associated degradation pathway [93]. Additionally, *TXN* has previously been shown to confer radio-resistance and mitigate radiation-induced lethality [94]. Similarly, *GSTP1* has been associated with increased radiation resistance [95] while *HMGB1* has been shown to promote cell survival and decrease apoptosis [96]. This highlights further genetic mechanism through which torpor may confer protective effects. 

### 3.5. Network Analysis of the Radiation and Torpor + Radiation Groups Reveal Differential Regulation of Radio-Resistant Genes

**Figure 10:**
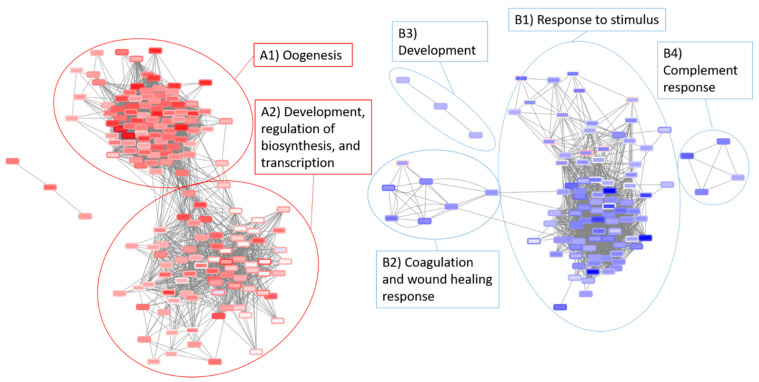
Integrated networks of the radiation (28.5-rad) and the torpor+radation group (18.5-mel-rad) generated by NetNC analysis. Fold change values for the radiation group are shown by node fill colour, values for torpor + radiation are represented by the shape border colour. Node shapes indicate: NetNC-predicted coherent genes in both groups (rectangular), in the radiation group only (oval), in the torpor + radiation group only (diamond) and changed in opposite directions (triangular). Results from each condition show very substantial overlap, with some notable differences being the upregulation of genes in the torpor + radiation group (18.5-mel-rad vs. 28.5-Ctrl) that confer radio-resistance (*TXN*, *GSTP1)* and promote survival (*HMGB1).*

NetNC [45] analysis of DE genes for the radiation (28.5-rad vs 28.5-Ctrl) and torpor + radiation (18.5-mel-rad vs 28.5-Ctrl) groups produced two networks for each condition (28.5-rad_NET_UP, 28.5-rad_NET_DOWN, 18.5-mel-rad_NET_UP, 18.5-mel-rad_NET_DOWN). Merging of the networks in Cytoscape produced an integrated network (as seen in Figure 10) (cytoscape network available in Supplementary Network S1) revealing considerable overlap in the NetNC results for each condition and producing two networks that reveal common response mechanisms. BiNGO [47] analysis of the merged upregulated clusters defined in comparison to the control group were annotated with GO terms and included oogenesis, development terms and the regulation of transcription, biosynthetic and metabolic processes. The merged downregulated clusters were annotated with a response to stimulus, coagulation, wound healing response, complement response and development. 

A comparison of networks across conditions was undertaken to inform the effects of torpor upon radiation response (Figure 10, Table 2). Four genes were present in 28.5-rad_NET_UP but absent from the NetNC results for 18.5-mel-rad (radNET_only, Table 2). These included *Mpz*, a myelin sheath component that is induced by axon injury [97], suggesting that torpor might protect against radiation-induced demyelination. A GABA symporter (*SLC6A1B*) important for gut motility and *Rho*, a GTPase involved in stress fibre formation [98,99] was also part of radNETonly. 

Thirteen genes showed opposite regulation profiles, occurring in both 18.5-mel-rad_NET_UP and 28.5-rad_NET_DOWN (OPP_13, Table 2). For example, *Mpx* upregulated in 18.5-mel-rad is a gene involved in myelopoiesis which is a process that has previously been reported to increase resistance to infection when induced prior to radiation [100]. OPP_13 also contained developmental (*HMGB3a*, *Foxj1b*, *tnni1b)* [101,102,103], proliferation (*epha4b)* [104] and, neuron development (*sulf2a*) [105] genes. Furthermore, we also observed upregulation of *Mylk*, which is important for gut motility [106] and epithelial survival [107] however as it also has roles in stress fibre formation [108] through rho signalling, further work should assess whether torpor regulates fibrosis. 

Pathway analysis of the radiation group suggests exposure reduces the availability of glucose in the GIT, whereas the NetNC results for the torpor + radiation group shows the upregulation of *ins*, involved in glucose uptake. Additionally, we also noted the upregulation of a fatty acid binding protein (*fabp7a*) expressed in CNS development [109] as well as the upregulation of *sst1.1* which has been observed to inhibit locomotion [110] and may therefore play a role in the reduced activity seen in the torpor model. 

Fourteen genes were present in 18.5-mel-rad_NET_UP and not in the NetNC results for the 28.5-rad (torpor_NET_only, Table 2). Several of these function in development, morphology (*wnt4, wnt11f2, irx2a, msx2b*, *sox19a*, *tbx5a)* [111,112,113,114,115,116] and cell proliferation *(msx2b*) [117]. Other genes belonging to torpor_NET_only function in neurogenesis *(Foxi1, irx2a, sox19a*) [118,119], neuron differentiation (*emx3, lmx1bb*) [120,121], synaptic function (*sncga*) [122] and plasticity (*pcp4a*) [123,124] which may be globally upregulated in response to the melatonin which has previously been associated with promoting neurogenesis [125,126]. Upregulation of stem cell, developmental and proliferative genes might offer protective affects by increasing cell survival and ensuring correct morphology during cell replacement responses, while those genes involved in neuronal development should be investigated further for signs of neuroprotective affects *in situ*.

## 4. Discussion

### 4.1. Induced Torpor Reduces Metabolism

Our work demonstrates a reduction in metabolism upon exposure to reduced ambient temperatures and melatonin as evidenced by the downregulation of key metabolic pathways namely, fatty acid degradation, glycolysis, oxidative phosphorylation and the TCA cycle. As reported in previous studies of cold acclimatisation, a hypometabolic state leads to an increase in energy stores. This has been associated with conferring a better physical condition, enabling cells to cope better with an increase in stress-associated energy demands and may therefore represent one way in which inducing torpor may confer protection against radiation stress [22]. Additionally, reduced temperatures have also been associated with a longer lifespan suggesting a slowing of the aging process in a hypometabolic state [127]. On the contrary, unsuccessful acclimatisation can cause thermal shock and cold stress leading to the depletion of lipid energy stores [128] as the main energy reserve mobilised during stress in zebrafish [129]. 

The torpor + radiation group also showed a downregulation of energy producing pathways including glycolysis, TCA cycle and oxidative phosphorylation while like that of the radiation group we see an increase in fatty acid metabolism. The induction of lipid metabolism is likely stress induced from radiation exposure; however, given that the experimental design included the use of torpor prior to radiation exposure which would increase in lipid stores, their depletion is less likely than without the use of torpor. Further work is needed to define energy expenditure and energy stores during torpor with the addition of stress. Furthermore, the suggested increase in lipid stores during torpor prior to radiation exposure is supported by and provides a rationale for the indications of ferroptosis, as an increase in lipid content would fuel an increase in peroxidation. While an increase in lipid energy stores leads to a better physical condition and by virtue a better ability to deal with stress, it is not yet known whether the benefits of increased energy supplies outweigh the increase risk of programmed cell death and this warrants further investigation.

### 4.2. Low Dose Radiation Perturbed Key Circadian Rhythm Genes

Our results suggest that low dose radiation causes perturbation of circadian rhythm given that all zebrafish were housed using the same light/dark cycle. Several factors associated with spaceflight influence circadian rhythms such as microgravity, lighting conditions, workloads and shift work [130]. However, while the effects of low dose radiation on circadian rhythm (CR) have not been well characterised in the context of spaceflight, this work suggests that radiation itself may be a contributing factor to its perturbation. Additionally, and more specifically, the CR also plays a role in the normal functioning of the GIT [131]. Circadian clock disruptions have been linked to various GIT-related disorders including constipation, IBS, peptic ulcers, metabolic syndrome or cancer [132]. These disorders alter the GIT functionality and represent major obstacles to maintaining astronaut health during long-term space travel. While these results reveal a radiation-induced increase in the CR gene, *Per3*, its expression in the torpor group was downregulated. Similarly, the absence of the enrichment of the CR in the torpor + radiation group suggests that its perturbation is not as pronounced during torpor which may mitigate its contribution to disease. Although, this may be a function of the addition of melatonin which may or may not feature in future models of induced torpor. Previous work has also shown that the CR can influence mouse GIT cell survival after radiation depending on when they were irradiated, which show increased apoptosis during waking hours (06:00–09:00) [133] and may suggest a therapeutic role for circadian altering drugs during solar particle events. Additional work is needed to further understand the role of circadian rhythm in the abnormal physiological effects of long duration spaceflight. 

### 4.3. Low Dose Radiation Induced a Glucocorticoid Stress Signalling Response

Our results indicate that radiation exposure is leading to glucocorticoid (GC) signalling which occurs in response to stress [134]. Given that our results represent a cell state 48 h after the last radiation exposure this suggests a prolonged stress response and is strengthened by the evidence showing that radiation produces a persistent stress response in space radiation studies of mouse intestine [135]. Sustained systemic GC levels have been implicated in pathologies such as osteoporosis, myopathy, reduced serum vitamin D levels and cataracts [136,137] which are already known to occur in spaceflight [138,139,140]. This points to a role for radiation-induced GC levels being a contributing factor. Furthermore, the link between GC signalling and the CR has long been known. The literature shows that rhythmic GC release is under circadian control via the hypothalamic-pituitary-adrenal axis [141], although, the expression of some peripheral clock genes are also induced by GCs (*PER2* for example) [142]. GCs are therefore considered to act as peripheral circadian co-ordinators [143]. These results therefore provide evidence that stress signalling can alter peripheral clock gene expression and provides a mechanism for radiation induced circadian perturbation in peripheral tissues such as the GIT. Given the wide-ranging roles in regulating homeostatic processes in the body and the disease-causing potential, further work should focus on the role of the CR and GC signalling during spaceflight.

### 4.4. Low Dose Radiation May Affect Nutritional Status of Astronauts

Nutrient uptake is an essential function of the GIT and its normal functioning during space travel is important to ensure the adequate nutritional status of astronauts during and post long-term missions. However, the GIT is a radiosensitive organ and space-related radiation and ROS is known to cause injury to the epithelial cells [135,144]. Radiation exposure has been observed to stimulate nutrient uptake by altering the expression of important nutrient transmembrane transport proteins in the gut epithelium [88]. Indeed, these results also show that a change in transporters in the GIT cells could affect gut functionality and the nutritional status of astronauts. For example, the increase in lipid uptake is consistent with a radiation-induced stress response given that lipids are the primary fuel source in times of stress in zebrafish [145]. Similarly, increased lipid and protein uptake may be a homeostatic measure to correct imbalances caused by ROS damage or replace energy stores depleted by a shift to their metabolism. The torpor + radiation group also had a dysregulation of genes involved in absorption and anion transport, which may suggest a lack of protection against changes to gut functionality. However, it has been suggested that future models of induced torpor in humans could deliver nutrients intravenously via Total Parental Nutrition, which is used in medical practice, contains all necessary nutrients and bypasses the digestive system mitigating the need for absorption in the GIT [146].

### 4.5. Torpor May Protect against Radiation through Increased Pro-Survival Signalling

One indication that torpor confers a radio-protective effect was the presence mitotic, pro-survival and anti-apoptotic signals. This feature is also backed by previous studies showing an increase in cell division in cold-acclimatised zebrafish attributed to the over-expression of *CDC48* [147]. These pro-survival signals were also apparent in the torpor + radiation group and were notable given that radiation is known to halt the cell cycle. Progression through the cell cycle might therefore indicate that compared to the radiation group, these cells received less damage, repaired it and overcame the cell cycle arrest phase. It might also be that an increase in mitogenic signals imparted from the torpor variable has swayed cell fate decisions to push through the cell cycle. It is important however to consider that this could lead to the passing on of harmful mutations to daughter cells, so more work is needed to define if there is a change in rate of mutations and diminished efficiency of repair mechanisms at colder temperatures. A summary table (Table S15) conveys the most notable and non-redundant biological themes impacted in each experimental condition. 

### 4.6. Torpor May Reduce Radiation-Induced Oxidative Stress

The radiation and the torpor + radiation group were both experiencing a stress response which was likely induced by radiation-induced ROS. This was indicated in the torpor + radiation group by the enrichment of glutathione metabolism, suggesting an antioxidant response. However this is not to say that oxidative stress is only occurring in this group. In fact, we posit that this group is experiencing less oxidative stress due in part to the reduction in metabolic pathways which generate ROS, and the ‘oxygen effect’. This phenomenon states that less radiation-induced ROS is generated by low oxygen concentrations in a cell, which normally positively correlates with the amount of damage received during irradiation [148]. Given that a hypoxic environment in the torpor + radiation group is supported by the enrichment of a response to decreased oxygen levels, radiation exposure in this group could be generating less ROS compared to the radiation-only group, at the same dose. The oxygen effect could therefore provide a plausible mechanism for a conferred radioprotective effect. In addition, the use of melatonin to increase sedation in this torpor model is also likely providing additive protection against the radiation exposure as an antioxidant which has previously been shown to attenuate gamma-ray-induced intestinal damage [149]. However, further functional work is needed to validate the results. 

### 4.7. Torpor May Lead to Removal of Radiation Damaged Proteins

Where one might expect an increase in protein degradation following radiation exposure previous work has shown counterintuitively, that low dose ionising radiation has an inhibitory effect on proteasome activity [150], explaining the absence of its enrichment in the results from the radiation group. In both the torpor-only and torpor + radiation group however, we see an increase in ER-associated protein processing pathways including ubiquitin-mediated degradation and refolding responses. The presence of this response in these groups suggests it is a response to reduced temperatures and is backed by studies in cold-adapted Antarctic fish showing elevated levels of protein ubiquitylation thought to protect cells from cytotoxic build-up of misfolded proteins [151,152]. We therefore suspect this to be the case in zebrafish, as a cold-tolerant species and suggest it may be beneficial in removing radiation-damaged proteins, although additional study is needed to assay the quality of cellular protein content.

### 4.8. Considerations for Inducing Torpor in Mammals

It has been suggested that zebrafish enable cold-tolerance through the production of specific isozymes through RNA processing and Spliceosome pathway (found to be enriched in our work) producing enzymes that are functional under colder temperatures [153,154,155]. Further work is needed to determine if this adaptive response to cold temperatures is conserved or needed in mammalian systems given that the induction of an appropriately designed synthetic torpor would mitigate the need to maintain normal physiological processes. 

Studies on the mechanisms of torpor induction in rodents have revealed biochemical controls of reducing metabolic rate by downregulating the electron transport chain, or a reduction in body temperatures via neuronal circuits in the hypothalamus in rodents [156,157]. In contrast studies into the induction of synthetic torpor in non-hibernating mammals have focused on molecules such as iodothyronamines [158], adenosine and α2 adrenergic agonists which confer metabolic suppressing traits [159]. While this study exploits the ability of zebrafish to reduce their metabolism and body temperatures in response to changing ambient temperatures [19], it is recognised that an important step to successfully inducing torpor in homoetherms will involve bypassing thermogenesis, a mechanism used to maintain a stable body temperature. Future research will therefore involve drugs that show promise in suppressing shivering such as meperidine (pethidine), clonidine and doxapram. These drugs are shown to be most effective without significant respiratory depression [21]. Further research will address how animals that utilise torpor have evolved to make it tolerable for long periods of time without sustaining injury, to elucidate the underlying mechanisms that would make it more tolerable in humans.

## 5. Conclusions

The reinvigoration of space exploration and interest in interplanetary manned missions to Mars will create both challenges and opportunities. For example, long-duration, Earth-independent missions will require sufficient food, water and oxygen to sustain a crew for long periods of time while maintaining quality and nutritional content, as well as exposing the crew to harmful galactic cosmic rays and potential solar flares. There is therefore an urgent need to develop innovative countermeasures to ensure crew health. This study showed that low dose radiation exposure, likely to be experienced on a 6-month journey to Mars can trigger a stress response involving, disruption of the circadian rhythm, cell cycle arrest, changes to metabolism and transporters that could impact nutrient uptake and digestion of lipids and proteins. However, an induced torpor state may confer protection by the induction of mitogenic, pro-survival signals, reducing apoptotic signals, lowering metabolism, increasing energy stores; enabling cells to meet energy demands. Similarly, a reduction in oxygen content in cells may decrease the amount of ROS that can be generated from the same dose of radiation, therefore leading to less cellular damage. While continuing work will validate the results, this detailed preview of the GIT transcriptome of an induced torpor model provides compelling evidence for a conferred radio-protective effect and promising clues for its exploitation during space travel.

## Figures and Tables

**Table 1:** Overview of the experimental groups showing the experimental group names, key, sample number per condition, values of radiation exposure, ambient water temperature and melatonin treatment.

Group	Key	Sample (N)	Radiation (cGy)	Water Temperature (°C)	Melatonin (µM)
Control	Ctrl	6	0	28.5	0
Melatonin	28.5-mel	6	0	28.5	24
Temperature	18.5-Ctrl	6	0	18.5	0
Torpor	18.5-mel	6	0	18.5	24
Radiation	28.5-rad	6	32.64	28.5	0
Torpor + radiation	18.5-mel-rad	6	32.64	18.5	24

**Table 2:** Genes found to be dysregulated between the radiation group and torpor + radiation group as determined from the integrated NetNC network.

radNET_Only	OPP_13	torpor_NET_Only
*rho* *mpz* *slc6a1b* *hoxb1b*	*mpx* *hmgb3a* *fabp7a* *myh7* *mylk* *ins* *epha4b* *sst1.1* *foxj1b* *tnni1b* *sulf2a* *actn2b* *sncga*	*msx2b* *lmx1bb* *wnt11f2* *myl7* *tbx5a* *smyd1b* *pomca* *sox19a* *wnt4* *gbgt1l4* *emx3* *pcp4a* *irx2a* *foxi1*

## Data Availability

The data that support the findings of this study are openly available in NCBI Gene Expression Omnibus, and are accessible through GEO Series accession number GSE169522 at https://www.ncbi.nlm.nih.gov/geo/query/acc.cgi?acc=GSE169522 accessed on 31 January 2021.

## Supplementary Materials

The following are available online at https://zenodo.org/record/4699873#.YH0YST8RVPY. Figure S1: iPG Metabolic pathways torpor vs control.png, Figure S2: IPG Protein processing in endoplasmic reticulum Torpor vs control.pn, Figure S3: iPG Metabolic pathways torpor with radiation vs control.png, Network S1: integrated network radiation vs control torpor with radiation vs control.cys, Video S1: Trial 1.mp4, Video S2: Trial 2.mp4, Video S3: Trial 3.mp4, Video S4: Trial 4.mp4, Video S5: Trial 5.mp4, Video S6: Trial 6.mp4, Video S7: Trial 7.mp4, Video S8: Trial 8.mp4, Video S9: Trial 9.mp4, Video S10: Trial 10.mp4, Video S11: Trial 11.mp4, Video S12: Trial 12.mp4, Table S1: Output Zebrafish Torpor Experiment, Table S2: qPCR primers.docx, Table S3: DEG torpor vs control group zebrafish and human IDs.xls, Table S4: webgestalt torpor vs control group zebrafish IDs.xlsx, Table S5: webgestalt torpor vs control human ID.xlsx, Table S6: ipathwayguide torpor vs control human IDs.xlsx, Table S7: DEG radiation vs control group zebrafish human IDs.xlsx, Table S8: webgestalt radiation vs control group zebrafish ID.xlsx, Table S9: webgestalt radiation vs control group human IDs.xlsx, Table S10: ipathwayguide radiation vs control group human IDs.xlsx, Table S11: DEG torpor with radiation vs control group zebrafish IDs human IDs.xlsx, Table S12: webgestalt torpor with radiation vs control zebrafish IDs.xlsx, Table S13: webgestalt torpor with radiation vs control human ID.xlsx, Table S14: ipathwayguide torpor with radiation vs control human IDs.xlsx, Table S15: impacted biological themes across each condition.docx.

## Abbreviations

DE	Differentially expressed
FC	Fold change
FTI	Functional Target Identification
CR	Circadian Rhythm
GC	Glucocorticoid
GIT	Gastrointestinal Tract
IACUC	Institutional Animal Care and Use Committee
IMP	Integrative Multi-species Prediction
MUSC	Medical University of South Carolina
ORA	Over-Representation Analysis
PCA	Principal component analysis
RAS	Renin-Angiotensin System
ROS	Reactive oxygen species
TCA	Tricarboxylic acid
